# Mesenchymal stem cell-derived exosomal microRNA-136-5p inhibits chondrocyte degeneration in traumatic osteoarthritis by targeting ELF3

**DOI:** 10.1186/s13075-020-02325-6

**Published:** 2020-10-27

**Authors:** Xue Chen, Yuanyuan Shi, Pan Xue, Xinli Ma, Junfeng Li, Jun Zhang

**Affiliations:** 1 Department of Orthopedics, The Second Hospital of Jilin University, No. 218, Ziqiang Street, Nanguan District, Changchun, 130041 Jilin Province People’s Republic of China; 2 Department of Nursing, The Second Hospital of Jilin University, Changchun, 130041 People’s Republic of China; 3 Intensive Care Unit, The Second Hospital of Jilin University, Changchun, 130041 People’s Republic of China; 4 Department of Clinical Laboratory, The Second Hospital of Jilin University, No. 218, Ziqiang Street, Nanguan District, Changchun, 130041 Jilin Province People’s Republic of China

**Keywords:** Traumatic osteoarthritis, Chondrocyte degeneration, Bone marrow mesenchymal stem cells, Exosome, microRNA-136-5p, ELF3, Extracellular matrix secretion, Migration

## Abstract

**Background:**

Emerging evidence suggests that microRNAs (miRs) are associated with the progression of osteoarthritis (OA). In this study, the role of exosomal miR-136-5p derived from mesenchymal stem cells (MSCs) in OA progression is investigated and the potential therapeutic mechanism explored.

**Methods:**

Bone marrow mesenchymal stem cells (BMMSCs) and their exosomes were isolated from patients and identified. The endocytosis of chondrocytes and the effects of exosome miR-136-5p on cartilage degradation were observed and examined by immunofluorescence and cartilage staining. Then, the targeting relationship between miR-136-5p and E74-like factor 3 (ELF3) was analyzed by dual-luciferase report assay. Based on gain- or loss-of-function experiments, the effects of exosomes and exosomal miR-136-5p on chondrocyte migration were examined by EdU and Transwell assay. Finally, a mouse model of post-traumatic OA was developed to evaluate effects of miR-136-5p on chondrocyte degeneration in vivo.

**Results:**

In the clinical samples of traumatic OA cartilage tissues, we detected increased ELF3 expression, and reduced miR-136-5p expression was determined. The BMMSC-derived exosomes showed an enriched level of miR-136-5p, which could be internalized by chondrocytes. The migration of chondrocyte was promoted by miR-136-5p, while collagen II, aggrecan, and SOX9 expression was increased and MMP-13 expression was reduced. miR-136-5p was verified to target ELF3 and could downregulate its expression. Moreover, the expression of ELF3 was reduced in chondrocytes after internalization of exosomes. In the mouse model of post-traumatic OA, exosomal miR-136-5p was found to reduce the degeneration of cartilage extracellular matrix.

**Conclusion:**

These data provide evidence that BMMSC-derived exosomal miR-136-5p could promote chondrocyte migration in vitro and inhibit cartilage degeneration in vivo, thereby inhibiting OA pathology, which highlighted the transfer of exosomal miR-136-5p as a promising therapeutic strategy for patients with OA.

## Background

Osteoarthritis (OA) is a common joint disease, which has increasing frequency in aging populations, thus bringing a considerable burden of disability [1]. The gradual loss of chondrocytes is the leading cause of OA since chondrocyte is the only cell constituting the articular cartilage [2]. However, the underlying molecular mechanisms of OA development remained enigmatic [3]. Though a recently reported study has provided the guideline for clinicians and patients to make treatment decisions for the management of OA [4], joint replacement surgery remained the most commonly applied treatment to date [5]. Therefore, there is a need to explore in greater detail the molecular mechanism underlying OA.

Exosomes are small (30–100 nm) membrane-bound extracellular vesicles that are released by various cells into biological fluid [6]. The primary function of exosomes is to act as an intercellular communicator shunting bioactive molecular cargo to target cells [7]. Mesenchymal stem cells (MSC)-derived exosomes have the potential to alleviate the pathological severity of OA. For example, the exosomes from embryonic stem cell-MSCs play a beneficial role in OA by balancing the synthesis and degradation of the chondrocyte extracellular matrix (ECM) [8]. In other contexts, MSC-derived exosomes have been reported to perturb the development of joint disease and repair damaged joints [9]. For example, treatment with MSC-derived exosomes has been indicated to repair temporomandibular joints and promote their regeneration in OA [10].

Accumulating studies have indicated the microRNAs (miRs) to be promising potential biomarkers for various diseases and specifically to play important roles in regulating major risk factors for OA [11, 12]. For instance, miR-29b-3p has been reported to improve the apoptosis of chondrocyte in OA by targeting PGRN [13]. Besides, the manipulation of the expression of miR-365 presents a potent therapeutic target for the prevention and treatment of osteoarthritis [14]. Moreover, intra-articular delivery of antago-mir-483-5p has been reported to inhibit OA and disturb its development by regulating the ECM enzymes matrilin 3 and tissue inhibitor of metalloproteinase 2 [15]. A recently reported study has indicated that silencing miR-136-5p effectively reduced the expression of inflammatory factors and chemokines to further protect the spinal cord via NF-κB/A20 signaling in vivo and in vitro [16]. Online targeting prediction software found that miR-136-5p could target E74-like factor 3 (ELF3), which belongs to the ETS family of transcription factors that participates in multiple activities of the progression of many diseases including inflammation and epithelial differentiation disorders [17]. Additionally, previous studies have also reported the elevated expression of ELF3 in cartilage tissues isolated from OA patients, which indicates the potential role of ELF3 as a central factor for cartilage degradation in post-traumatic OA in vivo [18]. Besides, the regulation of ELF3 expression by leptin could regulate the inflammatory responses induced by leptin in articular chondrocytes [19]. Hence, the present study aims to investigate the regulatory mechanism of MSC-derived exosomal miR-136-5p inhibiting chondrocyte degeneration of traumatic OA by targeting ELF3. Here, we attempted to highlight the significance and the need for further development on clinical therapy of OA.

## Methods

### Ethics statement

The current study was performed with the approval of the Ethics Committee of the Second Hospital of Jilin University. This study was according to the Helsinki Declaration, and all patients signed the informed consent with the approval of the review committee. All animal experiments were carried out according to the Animal Research: Reporting In-Vivo Experiments (ARRIVE) guidelines. The animal use program of this study was approved by the Animal Ethics Committee.

### Microarray-based gene expression analysis

ELF3 was selected for this study through microarray-based database screening. Six different databases including TargetScan (│Total context++ score│ ≥ 0.4) (http://www.targetscan.org/vert_71/), miRDB (Target Score ≥ 70) (http://www.mirdb.org/), RAID (Score > 0.2) (http://www.rna-society.org/raid2/index.html), miRWalk (│energy│ ≥ 18) (http://mirwalk.umm.uni-heidelberg.de/), StarBase (http://starbase.sysu.edu.cn/), and DIANA TOOLS (miTG score > 0.75) (http://www.microrna.gr/microT-CDS) were used to explore the upstream miRs of ELF3 and predict their regulatory pathways.

### Patient enrollment

A total of 20 cases of cartilage tissues from patients with traumatic OA of the knee and 18 cases of knee cartilage tissues from patients with post-traumatic amputation were collected from January 2015 to June 2018. Patients with rheumatoid arthritis and septic arthritis were excluded from this study. The enrolled 20 traumatic OA patients included 12 males and 8 females with an average age of 56.55 years, ranging from 47 to 69 years old. There were 18 cases of traumatic amputation, including 8 males and 10 females with an average age of 52.22 years, ranging from 42 to 66 years old.

### Isolation and identification of BMMSCs

The femur bone marrow isolated from the traumatized patients was aseptically collected into the heparin anticoagulation tube. Then, the bone marrow blood was centrifuged at 1500 rpm for 20 min and the fat layer was discarded and this step was repeated twice. Afterward, a total of 5 mL (density 1.077 g/mL) of the Ficoll separation solution was added to a new centrifuge tube, then and the diluted bone marrow solution was added to the upper layer of Ficoll separation liquid along the tube wall. The solution was then centrifuged at 2500 rpm for 20 min. The white cell layer was pipetted, resuspended, and centrifuged again to collect the pellets, which contain BMMSCs. After resuspending, the collected BMMSCs were subsequently cultured in DMEM/F-12 medium (Invitrogen Corp., Carlsbad, CA, USA) containing 10% fetal bovine serum (FBS). After 72 h, the medium and non-adherent cells were discarded followed by the addition of DMEM/F-12 complete medium for further culturing and passaging.

For osteogenic differentiation, the fifth generation of BMMSCs was cultured using osteogenic medium (Cyagen Biosciences Inc., Guangzhou, China). The cells were stained with alizarin red staining solution after 2 weeks. For adipogenic differentiation, cells were cultured using the StemPro® Adipogenesis Differentiation Kit (Gibco, Thermo Fisher Scientific Inc., Waltham, MA, USA) and stained with Oil Red O dye after 2 weeks. For chondrogenic induction, the synovial mesenchymal stem cells (SMSCs) were encapsulated in alginate gel beads and cultured using the StemPro® Chondrogenesis Differentiation Kit (Gibco, Thermo Fisher Scientific Inc., Waltham, MA, USA) and stained with the Acin blue dye after 4 weeks.

### Isolation and identification of exosomes

After achieving the 50–60% cell’s confluence, the BMMSCs were further cultured in the MesenGro® hMSC medium (StemRD, Burlingame, CA, USA) at 37 °C with 5% CO_2_ for 48 h. Conditioned medium (CM) was collected and centrifuged at 300×g for 15 min at 4 °C and then centrifuged at 2500×g for 15 min to remove dead cells and cell debris. After centrifugation, the supernatant was filtered through a 0.22 μM filter (Merck–Millipore, Darmstadt, Germany). The filtered solution was then transferred to a 15 mL Amicon Ultra-15 centrifugal filter unit (Merck-Millipore, Darmstadt, Germany) and centrifuged at 4000×*g* until the supernatant volume was concentrated to approximately 200 μL. The ultrafiltrate containing exosomes was placed on top of a 30% sucrose/D2O cushion and then transferred to a sterile Ultra Clear™ tube (Beckman Coulter, Brea, CA, USA). The ultrafiltrate was then ultracentrifuged at 100,000×*g* for 1 h at 4 °C. The pellet was resuspended in 15 mL PBS and centrifuged again at 4000×g to concentrate the volume to approximately 200 μL. The exosome particles were measured using the CD63 ExoElisa™ kit (System Biosciences, Palo Alto, CA, USA).

The size and distribution of exosomes were measured using the Nanosizer™ technology (Malvern Instruments, Malvern, UK) and analyzed using Zetasizer software (Malvern Instruments, Malvern, UK). The morphology of exosomes was observed by a transmission electron microscope. The exosomes were placed on a copper grid coated with formvar (Structure Probe, Inc., PA, USA). The grid was dried using 2% uranyl acetate and then observed using a Philips Morgagni 268D microscope (Philips, Amsterdam, Netherlands). The above-described procedures were repeated three times.

### Cell infection

To explore the function of miR-136-5p, the agomir, antagomir, and their negative controls were purchased from Ribbio (Guangzhou, China). Oligonucleotide transfection was performed with riboFECT™ CP reagent (RiboBio Co., Ltd., Guangzhou, China). To construct lentiviral vectors containing ELF3 overexpression, an oligonucleotide was synthesized before the purified PCR product was co-cleaved with the Xhol and MluI (PWPXL vector) and ligated with T4 ligase to construct a gene overexpression vector. The constructed core plasmid (16 μg) and two enveloped plasmids PSPAX2 (12 μg) and PMD2G (4.8 μg) were co-transfected into HEK293T cells on 6-well plates. The supernatant was collected 48 h post transfection and concentrated with Centricon Plus-70 filter (UFC910096, Millipore). The lentivirus with a titer adjusted to 10^8^ TU/mL was adopted in the experiment.

### Collection, isolation, and culture of primary chondrocyte

The cartilage was dissected from the subchondral bone and then detached with 4 mg/mL protease and 0.25 mg/mL collagenase P. The cells were cultured in DMEM/F-12 (Gibco, Thermo Fisher Scientific Inc., Waltham, MA, USA) medium containing 5% FBS (Gibco, Thermo Fisher Scientific Inc., Waltham, MA, USA) and 1% penicillin and streptomycin (Gibco, Thermo Fisher Scientific Inc., Waltham, MA, USA). The chondrocyte was used for experiments within 3–7 days.

### Chondrocyte endocytosis

The BMMSCs were detached with trypsin-EDTA and resuspended in 1 mL of MesenGro® hMSC medium. After the addition of 5 μL of Vybrant Dio solution (Molecular Probes, Carlsbad, CA, USA) to the suspension, the mixture was incubated for 15 min at 37 °C in 5% humidified CO_2_. The cells were then cultured in a complete medium until cell confluence reached about 50–60%. Exosomes were then isolated and cultured with chondrocytes for 6 h. After washing 3 times with PBS to remove free exosomes, cells were then fixed with 4% paraformaldehyde for 15 min and stained with 4,6-diamino-2-phenyl indole (DAPI) for 5 min. Finally, images were taken using a Leica DMI6000B fluorescence microscope (Leica Microsystems, Wetzlar, Germany).

### RNA isolation and quantitation

RNA was extracted from exosomes using the Total Exosome RNA Isolation Kit (Invitrogen, Carlsbad, CA, USA). The extracted RNA was subsequently used for the RT-qPCR assay. Total RNA was extracted from chondrocytes or synovial MSCs using the TRIzol reagent (Invitrogen, Carlsbad, CA, USA).

For miRNA, cDNA was synthesized using the human miR RT-qPCR examination kit (BioTNT, Shanghai, China) and RNU6B was used as an internal reference for miRNA.

For mRNA, cDNA synthesis was performed using a transscript® All-in-One-First-Strand cDNA synthesis supermix for RT-qPCR. RT-qPCR was performed using the TransStart® Top Green RT-qPCR SuperMix (Transgen Biotech, Beijing, China), and β-actin was used as an internal reference for mRNA. The sequences are presented in Table 1.

**Table 1:** Primer sequences for RT-qPCR

	Forward (5′-3′)	Reverse (5′-3′)
miR-136-5p	TCCGCCCCTCTAGTCGTGT	AAGGGAACAGGCGTGGACAG
ELF3 (human)	TGAACCTGCACACTCCAGTC	GGTTGCTCAGGGTCAGTACC
MMP-13 (human)	GCCTTCAAAGTTTGGTCCGATG	TGGTCAAGACCTAAGGAGTGGC
Collagen II (human)	CAGGACCAAAGGGACAGAAAGG	GCAGTTCACCAACCGTAGGAGT
Aggrecan (human)	AGCTCTGGGGAGGAATCTGG	GCAGTTCACCAACCGTAGGAGT
Sox9	GCTCTGGAGACTTCTGAACG	GGGTGGTCCTTCTTGTGCT
β-actin (human)	GATGTGGATCAGCAAGCAGG	AAAACGCAGCTCAGTAACAGTC
RNU6B	AACTCAAGACAATGGTGATAATGGT	AAAGAACAGAAAGGAATACGCAGA
ELF3 (mice)	CATCCTAATCCACCCCGAGC	GGCCTCTGAGCGAAGAAACT
MMP-13 (mice)	CTTCTTCTTGTTGAGCTGGACTC	CTGTGGAGGTCACTGTAGACT
Collagen II (mice)	CTGGTGGAGCAGCAAGAGCAA	CAGTGGACAGTAGACGGAGGAAAG
Aggrecan (mice)	CCTGCTACTTCATCGACCCC	AGATGCTGTTGACTCGAACCT
β-actin (mice)	CCTCTATGCCAACACAGT	AGCCACCAATCCACACAG

*RT-qPCR* reverse transcription quantitative polymerase chain reaction, *miR-136-5p* microRNA-136-5p, *ELF3* E74-like factor 3, *MMP-13* matrix metalloproteinase 13, *Sox9* 136-KB DEL, upstream regulatory region

### Western blot

Total protein was isolated from cells using radio-immunoprecipitation assay lysis (Beyotime, Shanghai, China). The protein sample was electrophoresed through a 10% sodium dodecyl sulfate-polyacrylamide gel and electrotransferred onto a polyvinylidene fluoride membrane. The membrane was then blocked in Tris-buffered saline with Tween 20 (TBST) containing 0.1% Tween 20 and 5% skim milk powder followed by incubation with primary and secondary antibodies. The primary antibodies were as follows: rabbit anti-CD63 (1:1000, ab134045), rabbit anti-CD9 (1:2000, ab92726), rabbit anti-CD81 (1:1000, ab109201), rabbit anti-Alix (1:1000, ab186429), rabbit Anti-Tsg101 (1:1000, ab30871), rabbit anti-MMP-13 (1:3000, ab39012), rabbit anti-collagen II (1:5000, ab34712), mouse anti-Aggrecan (1:100, ab3778), rabbit anti-Sox9 (1:1000, ab185230), rabbit anti-ELF3 (1:1000, ab194943), and rabbit anti-β-actin (1:1000, ab179467). The secondary antibodies were HRP-labeled rabbit anti-mouse immunoglobulin G (IgG) (1:5000, ab6728) and HRP-labeled goat anti-rabbit IgG (1:5000, ab6721). In our study, GAPDH was used as an internal reference for Western blot. Relative intensity of each band on Western blots was measured by compared with GAPDH content. Finally, staining was visualized with an enhanced chemiluminescence kit (Beyotime, Shanghai, China), and the specific bands were measured using a MicroChemi 4.2 system (DNR Bio-Imaging Systems, Jerusalem, Israel). All antibodies were purchased from Abcam Inc. (Cambridge, MA, USA).

### Chondrocyte migration by Transwell assay

After the chondrocytes were detached, 5 × 10^4^ cells were seeded in the apical chamber of a 24-well 8 μm aperture perforated plate (Corning Glass Works, Corning, N.Y., USA). A total of 600 μL of complete chondrocyte culture medium containing exosomes was added to the basolateral chamber of the Transwell plate and incubated at 37 °C for 12 h. The apical chamber was then fixed with 4% PFA for 15 min and stained with 0.5% crystal violet for 10 min. The surface of the apical chamber was wiped to remove cells that did not migrate to the surface of the basolateral chamber. Finally, five randomly selected regions were photographed and statistically analyzed using a Leica microscope (Leica Microsystems, Wetzlar, Germany).

### Dual-luciferase reporter assay

Wild-type (WT) and mutant-type (MUT) ELF3 plasmids were constructed to explore the targeting relationship between miR-136-5p and ELF3. Recombinant plasmids were verified by restriction enzyme digestion and DNA sequencing. HEK293T cells were added to 12 well plates. After 24 h, miR-136-5p agomir and ELF3 WT or ELF3 MUT were transfected with the cells by the Lipofectamine 2000 reagent. The samples were then co-transfected with a pRL-TK plasmid expressing Renilla luciferase. After 48 h of transfection, the cells were lysed. Firefly and Renilla luciferase activities were examined using a dual-luciferase reporter assay system (Biotek, Winooski, VT, USA).

### Mouse model of post-traumatic OA

A total of 48 healthy male C57BL/6 mice, aged from 5 to 8 weeks old, were randomly classified into 4 experimental groups: normal, post-traumatic oleanolic acid (OA), post-traumatic OA + exosomes, and post-traumatic OA + Exos^miR-136-5p^. Each group comprised of 12 mice, housed with two mice per cage. After the mice were anesthetized with isoflurane, the right ankle joint and knee joint of the mouse were placed in a custom processing platen in a mechanical test device. A single mechanical load was applied to the ankle joint ((1 mM/s to 12 N), causing the tibia to move forward relative to the femur and extended the anterior cruciate ligament beyond the point of failure. Immediately after the injury, the joint was injected with 100 μL of 10^11^ particles/mL exosomes and were allowed to recover from anesthesia before returning to their home cage. At 1 h after the injury, the mice were euthanized by inhalation of carbon dioxide and the joints were harvested [20]. All surface-soft tissues (skin, muscle, etc.) were removed to isolate cartilage tissues of joints.

### Safranin O/Fast Green staining

The obtained cartilage tissues were fixed in 4% PFA. The cartilage tissues were decalcified by placement in 10% EDTA for 4 weeks and cut into 4–5-μm-thick sections. Each section was embedded in paraffin and was then cut into 6-μm-thick sections for Safranin O/Fast Green staining.

### Immunohistochemistry

The obtained cartilage tissues from the joint were sectioned as above and treated with the following primary antibodies: rabbit anti-ELF3 (1:500, ab194943), rabbit anti-MMP-13 (1:200, ab39012), rabbit anti-Collagen II (1:200, ab34712), and mouse anti-Aggrecan (1:50, ab3778). All the antibodies were purchased from Abcam Inc. (Cambridge, MA, USA). Finally, ten randomly selected fields were examined under high magnification and analyzed and quantified using ImageJ software [21].

### Statistical analysis

All data were analyzed by the SPSS 21.0 software (IBM, Armonk, NY, USA). Measurement data were expressed as the mean ± standard deviation. Comparison between two unpaired sets of data with normal distribution and homogeneity of variance was analyzed using an independent sample *t* test. A comparisons among multiple groups were analyzed by one-way analysis of variance (ANOVA) followed by Tukey’s post hoc test. A value of *p* < 0.05 was regarded as statistically significant.

## Results

### ELF3 was a target gene of miR-136-5p and miR-136-5p was lowly expressed in cartilage tissues of traumatic OA

**Fig. 1:**
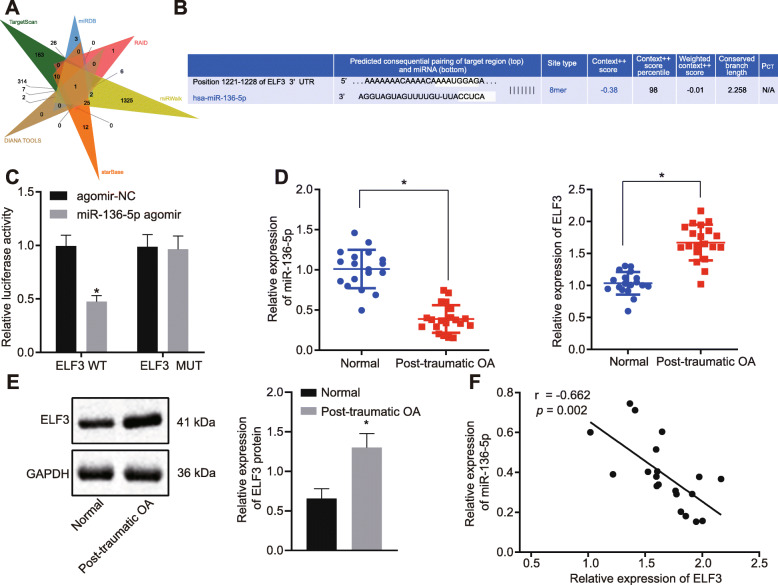
Overexpression of ELF3 and underexpression of miR-136-5p were observed in cartilage tissues of traumatic OA. **a** The Venn map of the upstream miR of the ELF3 by database search via in TargetScan, miRDB, RAID, miRWalk, starBase, and DIANA TOOLS. The only intersected miR was miR-136-5p. **b** Binding sites of miR-136-5p and ELF3 in TargetScan. **c** Dual-luciferase reporter assay of the miR-136-5p binding to ELF3. **d** The expression of miR-136-5p and ELF3 in normal cartilage tissues and traumatic OA cartilage tissues examined by RT-qPCR. **e** The expression of ELF3 in normal cartilage tissues and traumatic OA cartilage tissues examined by Western blot. **f** Pearson’s correlation analysis of the relationship between miR-136-5p and ELF3. Asterisk symbol indicated comparison with the normal group or the cells transfected with agomir-NC. A *p* < 0.05 was considered to be statistically significant. The above data were measurement data and expressed as the mean ± standard deviation. The independent sample *t* test was used for comparison between the two groups

Samples of cartilage tissues (obtained from patients with traumatic OA) exhibited a total of 650, 92, 17, 1796, 100, and 82 upstream miRs of ELF3 were obtained by screening six databases (TargetScan, miRDB, RAID, miRWalk, starBase, and DIANA TOOLS). miR-136-5p was found in the intersection of the Venn map (Fig. 1a). The map of binding sites (Fig. 1b) also defined the regulatory relationship between miR-136-5p and ELF3.

To further verify the targeting relationship between miR-136-5p and ELF3 in the cartilage tissues, dual-luciferase reporter assays were performed. We found that, by binding to wild-type 3′UTR, miR-136-5p transfection resulted in a decreased luciferase activity (indicating a decrease of ELF3 transcription), while the MUT 3′UTR sequence prevented the binding of miR-136-5p and ELF3 (Fig. 1c). To further explore the relevant mechanism, normal cartilage and traumatic OA cartilage samples were collected and then analyzed by RT-qPCR and Western blot to examine miR-136-5p and ELF3 expression in normal cartilage tissues and traumatic OA cartilage tissues. Our results (Fig. 1d, e) showed that ELF3 expression was increased in the traumatic OA cartilage tissues while miR-136-5p expression was reduced. Correlation analysis (Fig. 1f) revealed that miR-136-5p was negatively correlated with ELF3 expression. The above-described results indicated that ELF3 was a target gene of miR-136-5p and miR-136-5p had low expression in cartilage tissues of traumatic OA.

### miR-136-5p was expressed in both MSCs and their secreted exosomes

It has been reported in the literature that BMMSCs exosomes play a cartilage-protective role in OA [22]. Therefore, we further aim to explore whether the MSC-derived exosomes can be used as a delivery medium for miR-136-5p. For this purpose, human BMMSCs were first isolated and identified. We found that the cells were in a typical spindle type when the degree of cell confluence reached about 80–90% (Fig. S1A). Moreover, when BMMSCs were cultured in an osteogenic, adipogenic, or chondrogenic medium, BMMSCs were found to be easily induced to differentiate into OA, adipogenic, and chondrogenic directions (Fig. S1B). The above-described results indicated that the isolated cells were synovial MSCs with multipotential differentiation potential.

**Fig. 2:**
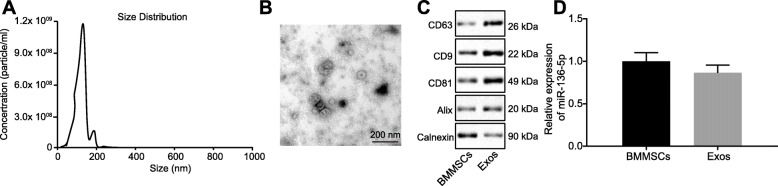
Identification of BMMSCs and exosomes. **a** DLS measuring the particle size and distribution of exosomes. **b** The morphology of exosomes (× 100,000) observed using a transmission electron microscope. **c** Western blot analysis of exosomes surface markers (CD63, CD9, CD81, and Alix) and endogenous proteins (TSG101). **d** The expression of miR-136-5p in BMMSCs and their secreted exosomes examined by RT-qPCR. The above data were measurement data and expressed as the mean ± standard deviation. The independent sample *t* test was used for comparison between the two groups

**Fig. 3:**
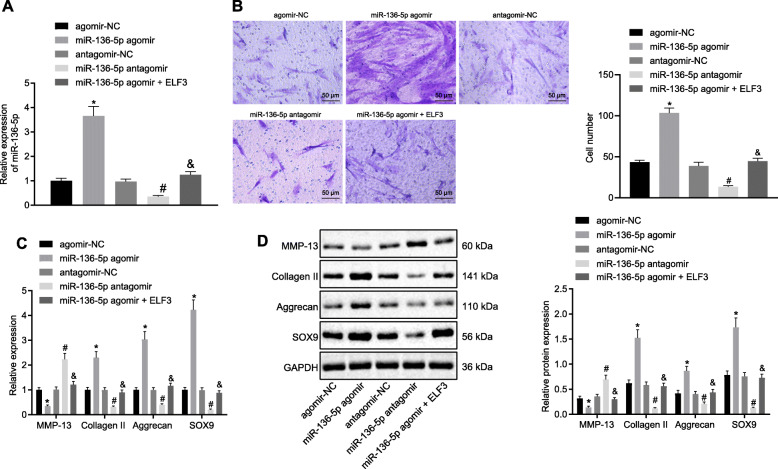
The migration and ECM secretion of chondrocytes were promoted by miR-136-5p through ELF3. **a** The expression of miR-136-5p in transfected chondrocytes examined by RT-qPCR. **b** Migration analysis of chondrocytes (× 200). **c** The mRNA expression of MMP-13, collagen II, aggrecan, and SOX9 in chondrocytes examined by RT-qPCR. **d** The protein expression of MMP-13, collagen II, aggrecan, and SOX9 in chondrocytes examined by Western blot. Asterisk symbol indicated *p* < 0.05 compared with the cells transfected with agomir-NC. Number sign indicated *p* < 0.05 compared with the cells transfected with antagomir-NC. Ampersand symbol indicated *p* < 0.05 compared with the cells transfected with miR-136-5p agomir. *p* < 0.05 indicated a significant difference. The above data were measurement data and expressed as the mean ± standard deviation. The data among groups were compared using one-way ANOVA: followed by Tukey’s post hoc test

Exosomes were isolated from conditioned medium containing BMMSCs. The secreted particles of BMMSCs were characterized by the dynamic light scattering (DLS), transmission electron microscope, and Western blot analysis. The results (Fig. 2a) showed that most vesicles were detected in size ranging from 50 to 150 nm. These hollow spherical microvesicles (Fig. 3b) further confirmed the expression of the abundant exosome markers: CD63, CD9, CD81, and Alix by Western blot, but not the endogenous protein TSG101 (Fig. 2c). Collectively, these results indicated that the exosomes were successfully isolated, while RT-qPCR was used to examine the miR-136-5p expression in cells and exosomes and the results (Fig. 2d) demonstrated that miR-136-5p was expressed in both MSCs and their secreted exosomes.

### miR-136-5p promotes the progression of chondrocytes and the secretion of ECM by ELF3

To further explore the function of miR-136-5p, chondrocytes were isolated from clinical normal cartilage tissues and subjected to miR-136-5p and ELF3 transfection (Fig. 3a). Transwell experiment was performed to examine the migration ability of cells. Our results exhibited a significant increase in the number of migrated cells in the cells transfected with miR-136-5p agomir in comparison with the cells transfected with agomir-NC, whereas it was reciprocal in the cells transfected with miR-136-5p antagomir compared with the cells transfected with antagomir-NC. The number of migrated cells in the cells transfected with miR-136-5p agomir and ELF3 chondrocytes was significantly reduced when compared to the miR-136-5p agomir group (Fig. 3b).

RT-qPCR and Western blot demonstrated that the content of collagen II, aggrecan, and SOX9 were significantly increased in the chondrocytes of the cells transfected with miR-136-5p agomir, whereas the content of MMP-13 was decreased compared with the cells transfected with agomir-NC. Besides, the content of collagen II, aggrecan, and SOX9 were decreased in the chondrocytes of the cells transfected with miR-136-5p antagomir, and the content of MMP-13 was increased when compared with the cells transfected with antagomir-NC. The levels of collagen II, aggrecan, and SOX9 were decreased in the cells transfected with miR-136-5p agomir; however, the level of ELF3 and MMP-13 was increased when compared with the cells transfected with miR-136-5p agomir (Fig. 3c, d). Collectively, the above-described results indicated that miR-136-5p could promote the migration and ECM secretion of chondrocytes by ELF3.

### Exosomal miR-136-5p promotes the migration and ECM secretion of chondrocytes

**Fig. 4:**
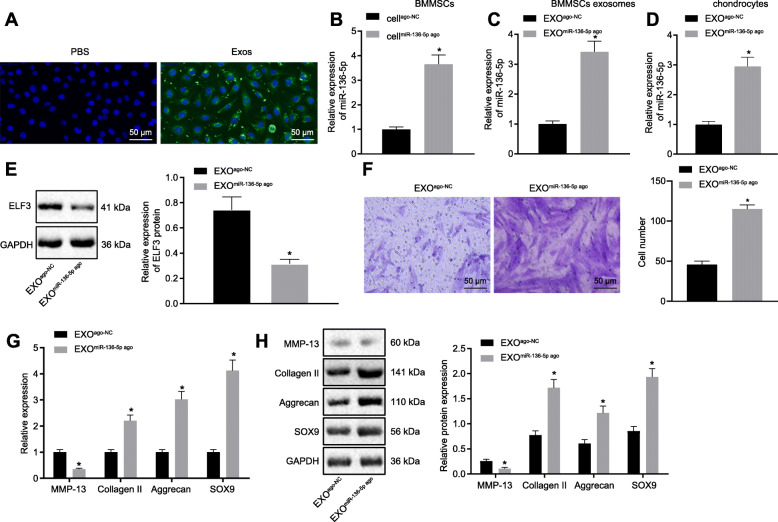
The migration and ECM secretion of chondrocytes were promoted by miR-136-5p-exo. **a** A typical immunofluorescence pattern of DIO (green)-labeled exosomes absorbed by chondrocytes, whose nuclei were stained with DAPI (blue) (× 200). **b** The expression of miR-136-5p in transfected BMMSCs examined by RT-qPCR. **c** The expression of miR-136-5p in exosomes secreted from transfected BMMSCs examined by RT-qPCR. **d** The expression of miR-136-5p in chondrocytes after exosome treatment examined by RT-qPCR. **e** Western blot analysis of the expression of ELF3 in chondrocytes treated with exosomes. **f** Migration analysis of chondrocytes (× 200). **g** The mRNA expressions of MMP-13, collagen II, aggrecan, and SOX9 in chondrocytes examined by RT-qPCR. **h** Western blot analysis of the protein expressions of MMP-13, collagen II, aggrecan, and SOX9 in chondrocytes. Asterisk symbol indicated comparison with the EXO^ago-NC^ or cell^ago-NC^ group. A value of *p* < 0.05 was considered to be statistically significant. The above data were measurement data and expressed as mean ± standard deviation. The independent sample *t* test was used for comparison between the two groups. One-way ANOVA was used among multiple groups, followed by Tukey’s post hoc test

BMMSCs were labeled with green fluorescent lipophilic dyes (Vybrant DiO) before the acquisition of exosomes to verify whether BMMSCs exosomes can serve as a carrier of miR-136-5p, as well as whether chondrocytes can absorb exosome-derived from BMMSCs. The chondrocytes were incubated with the exosomes from the labeled cells for 6 h, while DIO-labeled exosomes were observed in the perinuclear region of chondrocytes (Fig. 4a), indicating that chondrocytes can internalize exosomes. Besides, we used exosomes derived by BMMSCs to treat the chondrocytes, followed by RT-qPCR detection for miR-136-5p expression. As shown in Fig. S2, relative to the chondrocytes without exosome treatment, the chondrocytes incubated with BMMSC-secreted exosomes exhibited increased expression of miR-136-5p. Then, BMMSCs were subjected to miR-136-5p overexpression (Fig. 4b) and the exosomes were isolated for subsequent experiments.

The results of RT-qPCR (Fig. 4c) exhibited the upregulation in the expression of miR-136-5p in the secreted exosomes of BMMSCs that has been subjected to the overexpression miR-136-5p. Moreover, the expression of miR-136-5p in chondrocytes was further examined after internalization. The results (Fig. 4d, e) showed that the expression of miR-136-5p was increased in chondrocytes after endocytosis, whereas the expression of ELF3 was decreased. The chondrocytes were then treated with the abovementioned isolated exosomes followed by examining the migration ability of the cells by Transwell assay. The results (Fig. 4f) showed that notably higher numbers of migrated cells in the cells transfected with EXO^miR-136-5p ago^ group than in the cells transfected with EXO^ago-NC^. RT-qPCR and Western blot (Fig. 4g, h) showed that collagen II, aggrecan, and SOX9 levels were increased in the chondrocytes of the cells transfected with EXO^miR-136-5p-^ compared with the cells transfected with EXO^ago-NC^, whereas MMP-13 expression was decreased.

### EXO^miR-136-5p^inhibits cartilage degradation

**Fig. 5:**
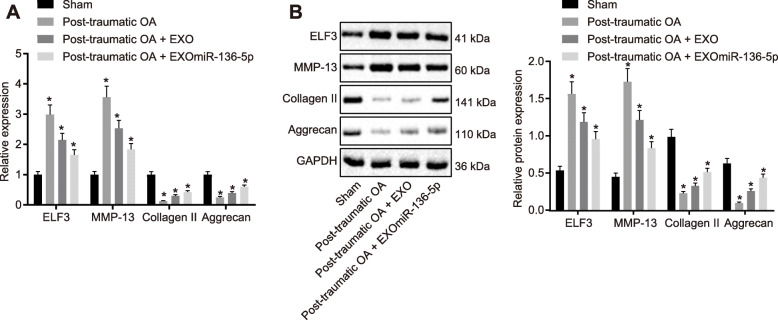
EXO^miR-136-5p^ inhibited cartilage degradation. **a** RT-qPCR examining the expressions of ELF3, collagen II, aggrecan, and MMP-13 in cartilage tissues of mice in each group. **b** Western blot examination of the expressions of ELF3, collagen II, aggrecan, and MMP-13 in cartilage tissues of each group. Asterisk symbol indicated comparison with the mice received sham operation. A value of *p* < 0.05 was considered to be statistically significant. The above data were measurement data and expressed as mean ± standard deviation. Data comparison among groups was performed using one-way ANOVA, followed by Tukey’s post hoc test

To further investigate the role of exosomal miR-136-5p in OA, a mouse model of traumatic OA was established and used for testing the prophylactic potential of exosomal miR-136-5p in traumatic OA (Fig. S3A-B). The cartilage matrix of the post-traumatic OA group was reduced; collagen II and aggrecan expressions were decreased in the damaged cartilage while ELF3 and MMP-13 expressions were upregulated compared with the mice that received sham operation. The loss of cartilage matrix in the post-traumatic OA mice injected with EXO was lesser than that in the post-traumatic OA mice. However, the cartilage matrix of the post-traumatic OA mice injected with EXO^miR-136-5p^ was still lost, but the severity was lesser than that of the post-traumatic OA mice or the post-traumatic OA mice injected with EXO. The expression of collagen II and aggrecan was slightly lower than that of the normal group but higher than that in the post-traumatic OA mice or the post-traumatic OA mice injected with EXO group. These tissues staining results were consistent with mRNA and protein expression of ELF3, MMP-13, collagen II, and aggrecan (Fig. 5a, b). Collectively, these results indicated that exosomal miR-136-5p could inhibit early post-traumatic OA and prevent further damages to the knee cartilage.

## Discussion

OA remains the most common inflammatory joint disease, which adversely affects the mobility of aging populations [23], now affecting perhaps 240 million individuals across the world [24], while age-related cumulative tissue damage resulting from mechanical erosion is considered as the leading cause of OA [25]. Unfortunately, to date, no effective method for early diagnosis of OA is available [26], yet current therapies for OA often pose their side effects or require invasive surgery [27]. The present study attempted to provide evidence that miR-136-5p derived from MSCs could inhibit the chondrocyte degeneration of traumatic OA by targeting ELF3.

The major findings of our study revealed that the ELF3 gene was highly expressed while miR-136-5p exhibit lower expression in the cartilage tissue of traumatic OA. ELF3 has been reported to act as an inflammatory mediator to destruct cartilage in OA [19]. Importantly, ELF3 has indicated being involved in the progressions of many diseases, including OA. For instance, increased expression of ELF3 was observed in human cartilage of OA patients [18]. Besides, dysregulation of ELF3 in OA disease has been illustrated to enhance the cartilage catabolism and abnormal anabolism in the damaged cartilage [28], though ELF3 and HMGA1, both transcription factors, have been reported to play an important role in the homeostasis of articular cartilage in OA [29]. However, ELF3 possesses the ability to regulate some inflammatory mediators in chondrocytes [28]. Importantly, the present study revealed the presence of over-expression of ELF3 in the cartilage tissues of traumatic OA patients and the mouse model.

On the other hand, miRs are endogenous non-protein-coding RNA molecules with a crucial role in regulating the expression of the post-transcriptional gene [30]. Emerging evidence has shown that the correlation between miRs and the pathogenesis of OA [31]. For instance, a decreased level of miR-136 was observed in the plasma of patients with knee OA [32]. Another study demonstrated that SNHG14 could promote an inflammatory response by increasing the expression of ROCK1 while decreasing miR-136-5p expression in oxygen-glucose deprivation and reoxygenation induced damage [33]. Moreover, miR-136 has been reported to improve neurocyte apoptosis associated by spinal cord ischemia injury by mediating changes in TIMP3 expression [34]. Consistently, our results revealed that miR-136-5p was downregulated in cartilage tissues of traumatic OA.

Moreover, we found that miR-136-5p could remarkably promote the migration of chondrocytes and ECM secretion by ELF3. Similar to our findings, it has been demonstrated that the binding of miR-320a-3p to the 3′UTR region of ELF3 could transcriptionally inhibit the expression of ELF3 [35]. Meanwhile, miRs have indicated to be associated with the migration of chondrocytes. For example, the over-expression of miR-195 could regulate the migration of chondrocyte by targeting GIT1 [36], while over-expressed miR-486-5p was capable of suppressing the migration of chondrocytes by inhibiting SMAD2 [37]. Additionally, it has been reported that miR-139 could inhibit the migration of chondrocytes [38]. Herein, we further verified that miR-136-5p could promote the migration of chondrocytes and conversely that ELF3 could inhibit the migration of chondrocytes. However, the extracellular matrix (ECM) is a complicated meshwork that is comprised of structural proteins and glycosaminoglycans [39]. Moreover, dysregulation of ECM is attributed to the development of various diseases, thus indicating the importance of maintaining the correct biochemical and biophysical properties of ECM [40]. Accordingly, the present study showed that miR-136-5p could promote the secretion of ECM, which could be inhibited by ELF3. Additionally, previously reported studies have demonstrated that the degeneration of articular cartilage contributes to the development of OA.

Further studies have illustrated the chondrocyte hypertrophy as a critical and final step in chondrocyte differentiation and OA pathogenesis [41]. However, the low accumulation of collagen in the ECM has been considered as a critical problem in damaged cartilage tissues [42], while proteoglycan aggrecan plays an essential role in the ECM growth plate cartilage [43]. Intriguingly, the transcription factor SOX9 has been reported to be a key regulator in the development of chondrocytes [44]. Yet, MMP-13 plays a central role in mediating the degradation of ECM components [45]. In accord with these above-described studies, our present data revealed significantly increased levels of collagen II, aggrecan, and SOX9, while there was a dramatic reduction in the MMP13 expression upon overexpression of miR-136-5p. Taken together, over-expression of miR-136-5p could inhibit cartilage degeneration.

Of note, exosomes are small membrane-enclosed vesicles that functioned as signaling mediators between cells [46], by delivering miRNAs and other bioactive to the target cells [47], while exosomes have been indicated to affect the migration of chondrocytes. For example, synovial MSC-derived exosomal miR-140-5p could also promote the migration of articular chondrocytes [48]. Inconsistent with these previously reported studies, our results revealed that exosomal miR-136-5p could promote the proliferation and migration of chondrocytes by targeting ELF3.

## Conclusions

**Fig. 6:**
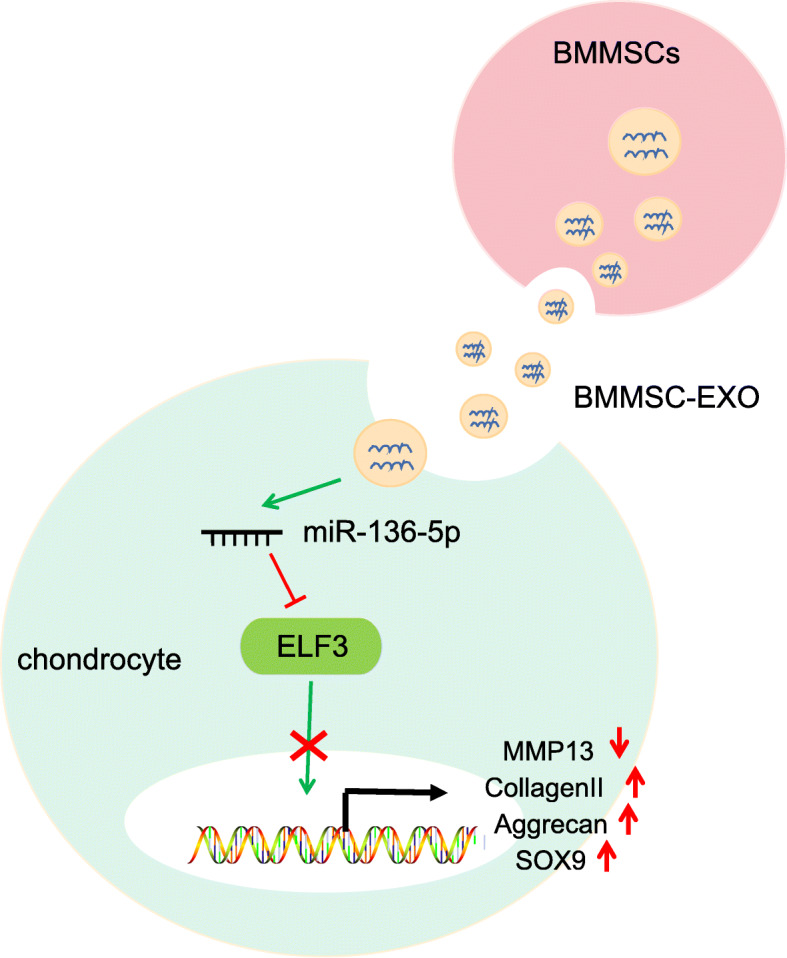
Schematic diagram illustrating that BMMSCs exosomal miR-136-5p could promote chondrocyte migration and ECM secretion by targeting ELF3, thereby inhibiting cartilage degradation in traumatic OA

Our study exhibited a significant reduction in the miR-136-5p expression and an increase in the ELF3 level in the cartilage tissues of traumatic OA patients and model mice. Moreover, we also unraveled that exosomal miR-136-5p was endocytosed in chondrocytes, which decreased the expressions of ELF3 and MMP-1 were decreased and increased levels of collagen II, aggrecan, and SOX9, thereby promoting the migration of chondrocytes and inhibiting cartilage degeneration (Fig. 6). Hence, this study provides insights into the development of a novel therapeutic target for treating OA, pending a more detailed validation eventually leading to clinical trials.

## Supplementary information

**Additional file 1: Fig. S1.** Identification of BMMSCs and exosomes. A: BMMSCs exhibiting a typical spindle-like morphology (100 ×). B: The pluripotent differentiation ability of BMMSCs in osteogenesis (left: 200 ×): adipogenic (middle: 200 ×) and chondrogenesis (right: 200 ×).

**Additional file 2: Fig. S2.** Expression of miR-136-5p in the chondrocytes with or without treatment with BMMSCs-serived exosomes; * indicated *p* < 0.05 compared with the chondrocytes without treatment with BMMSCs-serived exosomes (control).

**Additional file 3: Fig. S3.** EXO^miR-136-5p^ inhibited cartilage degradation. A: Cartilage tissues of each group were subjected to Safranin O/Fast Green staining morphology analysis (× 100). B: Immunohistochemical analysis (× 200) of cartilage tissues in mice of each group.

## Data Availability

The datasets generated and/or analyzed during the current study are available from the corresponding author on reasonable request.

## Abbreviations

BMMSCs: Bone marrow mesenchymal stem cells
OA: Osteoarthritis
ECM: Extracellular matrix
miRs: microRNAs
ELF3: E74-like factor 3
ARRIVE: Animal Research: Reporting In Vivo Experiments
SMSCs: Synovial mesenchymal stem cells
EDTA: Ethylenediaminetetraacetic acid
CM: Conditioned medium
PFA: Paraformaldehyde
WT: Wild-type
MUT: Mutant-type
